# Yoga for older adults with multimorbidity: teaching insights for optimising participant safety and inclusion from the process evaluation of the Gentle Years Yoga trial

**DOI:** 10.1136/bmjopen-2024-097472

**Published:** 2025-06-27

**Authors:** Lesley Ward, Laura Wiley, Fiona Rose, Jenny Howsam, Laura Bissell, Garry Tew, Tim Rapley

**Affiliations:** 1 Department of Social Work, Education and Community Wellbeing, Northumbria University, Newcastle upon Tyne, UK; 2 York Trials Unit, University of York, York, UK; 3 British Wheel of Yoga Qualifications, London, UK; 4 Institute for Health and Care Improvement, York St John University, York, UK

**Keywords:** Multimorbidity, Aging, COMPLEMENTARY MEDICINE, Exercise, QUALITATIVE RESEARCH, Self-Management

## Abstract

**Objectives:**

To develop a teaching exemplar for optimising the safe and accessible delivery of chair-based yoga to multimorbid older adult populations.

**Design:**

A qualitative process evaluation embedded within the multi-site, randomised controlled Gentle Years Yoga trial for older adults (65+ years) with two or more long-term health conditions (trial status: completed).

**Setting:**

Online and face-to-face interviews were conducted with participants and yoga teachers involved in the 12-week chair-based yoga intervention. Interview data were supplemented with observations of in-person and online yoga class delivery.

**Participants:**

All yoga teachers delivering the yoga intervention were invited to take part in the interviews, together with a subsample of participants receiving the yoga intervention. Participants were purposively selected to represent the trial cohort demographics of gender, age, ethnicity, index of multiple deprivation, and number and intensity of chronic health conditions.

**Results:**

25 yoga participants and 11 yoga teachers took part in one (N=19) or two (N=17) interviews. Participants were aged 66–91 years (mean age 74 years), with 2–8 long-term health conditions, most commonly osteoarthritis (N=15, 60%), cardiovascular disease (N=14, 56%), sensory conditions (N=9, 36%) and depression or anxiety (N=8, 32%). Yoga teachers were predominantly female (N=10, 91%), with 4–35 years yoga teaching experience across multiple yoga styles. Feedback from yoga teachers and participants was classified into six categories, generating a 21-item teaching exemplar. These covered aspects of delivery including class size and delivery formats, choosing appropriate physical content, enhancing inclusivity of personal beliefs through non-physical content, proactive teaching styles, communication tips and ways to boost visibility.

**Conclusions:**

This 21-item list adds to the current educational base of yoga for older adults. Addressing both face-to-face and online class formats, this exemplar offers pragmatic guidance for yoga teachers to enhance the safe and accessible delivery of chair-based yoga to older adults and multimorbid populations.

**Trial registration number:**

ISRCTN13567538.

**STRENGTHS AND LIMITATIONS OF THIS STUDY**

- A robust process evaluation embedded within a large-scale multisite randomised controlled trial.
- Pragmatic teaching exemplar based on the perspective of stakeholders engaged in both the delivery and practice of yoga for older adults with multiple long-term health conditions.
- Provides guidance for both in-person and online delivery of yoga classes.
- Findings are focused on chair-based yoga delivery.

## Introduction

 The older adult population (60 years and over) is projected to double globally by 2050.^1^ Parallel with an ageing population are issues associated with multimorbidity, the presence of two or more long-term health conditions^2^ and concomitant polypharmacy.^3^ As people live longer, and with more health conditions, the use of non-pharmacological treatment options and actively engaging individuals in their health management are recommended.^4 5^

Yoga is an increasingly popular form of health management. Originally a spiritual practice for uniting the body, mind and emotions,^6^ yoga was first recorded over 4000 years ago as one of six fundamental systems of Indian philosophy. The traditional practice of yoga consists of eight limbs, or paths, including ethical (Yama) and moral (Niyama) guidance, physical postures (Asana), breathing practices (Pranayama) and mental practices such as directed awareness (Pratyahara) and focused concentration (Dharana).^7 8^

Yoga has proliferated in the West over the past two decades.^9^ There are many styles of yoga,^10^ each with their own emphasis on, and delivery of, the components of the eight limbs. In the West, the more familiar form is Hatha yoga,^11^ a combination of physical, breathing and mental practices. Surveys suggest the average yoga user is single, white/European, college educated and female,^9 12 13^ with users most typically citing musculoskeletal and mental health management as reasons for practising yoga.^12^

However, a recent shift in demographics has shown increasing popularity for yoga as a form of complementary therapy among older adults.^14^ Evidence suggests differences in age-associated motivations for beginning yoga, with adults over 55 years more likely to practice yoga for managing health issues such as osteoporosis and those under 55 years more likely to engage in yoga for weight management and strength building.^15^ Pathways to yoga also show age-related differences, with older adults more influenced by social and community networks and younger adults more influenced by media.^15^

The increased use of yoga for health management parallels a strengthening evidence base for the effectiveness of yoga across a range of physical and mental health conditions, many of these common to older adults. These include cardiovascular disease,^16^ anxiety,^17^ cognitive function,^18^ type 2 diabetes,^19^ osteoarthritis^20^ and low back pain.^21^ Evidence further suggests yoga as a safe and acceptable practice for this age cohort.^22^,^24^

Despite this strengthening picture of the safety and effectiveness of yoga as a health management tool for older adults, and recommendations for yoga instructors and studios to target a broader demographic independent of age,^15^ yoga teacher training courses are mostly unregulated, and targeted towards teaching a more able-bodied demographic. Research is addressing this knowledge and skills gap, with several informative publications available on the adaptation of yoga postures to reduce risk and enhance physical outcomes in older adults.^25^,^28^

Content of yoga interventions for older adults commonly focus on improving strength, balance and mental focus,^25^ and targeting movements used in activities of daily living.^26^ Furthermore, there is a focus on adapting physical content to reduce risks associated with age-related conditions such as cardiovascular disease and osteoporosis,^27^ through enhancing core stability, accommodating restrictions in range of motion and protecting spinal health.^25^

Guidance to date tends to focus on the more typical mat-based delivery of yoga, and a gap remains for those teaching more accessible forms such as chair-based yoga. This paper adds to the current educational base by addressing calls for guidance to yoga teachers on optimising delivery of yoga for an older adult cohort.^24^

### The Gentle Years Yoga trial

Full details and associated outcomes of the Gentle Years Yoga (GYY) trial are reported elsewhere.^29^,^31^ Briefly, the GYY trial was a multisite, randomised controlled trial with embedded process and economic evaluations. The trial investigated the impact of a 12-week GYY programme compared with usual care on health-related quality of life and a range of secondary psychosocial health outcomes in multimorbid older adults (65 years and over) recruited from 15 primary care practices across England and Wales. Intervention participants received a weekly 75 min chair-based yoga class comprising postural, breathing and awareness practices adaptable to the physical and cognitive ability of attendees.^32^ Classes were followed by an optional 15 min social time. Yoga teachers delivering the trial intervention were qualified GYY teachers and received additional trial-specific training comprising a training day and delivery manual. Teachers devised their own class content, subject to preapproval by two GYY trainers to ensure the yoga practices aligned with GYY principles and adhered to a standardised class format. The group-based programme was initially delivered face-to-face, transitioned to online delivery via Zoom in response to COVID-19 social restrictions and was delivered in both formats for the final phase of the trial.

Quantitative findings indicated that while the GYY programme was not associated with any statistically significant benefits in health-related quality of life or secondary psychosocial health outcomes, it was safe and acceptable to the older adult cohort.^30^ The process evaluation further indicated the impact of GYY yoga ranged from minimal to transformative, dependent on meaningful improvements in biopsychosocial health^33^ and that online delivery was both an acceptable and often preferred form of accessing the yoga classes.^34^

### Aim

The aim of this paper was to formulate a teaching exemplar for face-to-face and online delivery of chair-based yoga to multimorbid and older adult populations, based on the experiences of yoga teachers and participants from the GYY trial. This combined stakeholder perspective serves to advance the knowledge base and role of yoga as a health-management tool for older adults with multimorbidity.

## Methods

### Study design of the GYY process evaluation

This qualitative study formed part of the embedded process evaluation of the GYY trial and was designed, analysed and reported according to robust qualitative research standards.^35 36^

One-to-one interviews and yoga class observations were conducted; any non-interview participants who declined consent for observation were excluded from observation field notes. Interviews were initially conducted face to face, then via Zoom or telephone following COVID-19 social restrictions, from October 2020 to April 2022. No other persons were present during the interviews except the researcher and participant. Interviews occurred across and following delivery of the 12-week yoga programmes, with a subset of participants and teachers invited to a second (longitudinal) interview. One participant declined a second interview, as they felt they had no further insights to add. These broad time periods intentionally captured both real-time and reflective experiences of the GYY trial programme.

### Patient and public involvement

Patient and public involvement representatives were involved in the GYY trial from grant preparation through to dissemination, to ensure the study design and outcome dissemination would be meaningful and relevant to an older adult, multimorbid population.

### Recruitment

To gain a broad perspective of optimal teaching techniques for delivery of yoga to this older age cohort, both trial teachers and participants were recruited. All 11 yoga teachers delivering the yoga programme were invited to take part in the interviews. Participant recruitment occurred in parallel with iterative data analysis, with sampling continuing until meaning saturation of the data occurred.^37^ A sample of 25 participants was recruited; participants were purposely selected to represent the trial cohort demographics of gender, age, ethnicity, index of multiple deprivation (IMD), and number and intensity of chronic health conditions.

### Data collection

Data were collected via individual interviews (audio recordings) and class observations (note taking), conducted by a university-based researcher (LWa) with over 10 years qualitative research experience in the fields of yoga and health. Interviews were semistructured, with interview schedules specifically developed for this project and iteratively adapted in response to cumulative interview and observational data. To compare yoga teacher and participant perspectives, questions were similarly worded where possible (box 1). Interviews were audio recorded, independently transcribed verbatim and anonymised by removing any reference to individuals or delivery site. Participants were offered the opportunity to provide feedback on and approve their anonymised transcripts; none requested this option. Observation field notes were written in real time to prevent recall bias, and additional field notes were written after each interview and observation. These notes were used to further tailor the interview schedules regarding the delivery aspects of the yoga classes.

Box 1 **Exemplar of core semistructured interview questions**

**Sample question (wording adapted to suit interviewee)**

- How has your experience of the yoga classes changed over the sessions you have delivered so far? Prompt for any practical problems faced.
- How well do you feel participants have engaged with the classes now? Prompt for what aspects have worked well/not so well. Prompt for what participants seem to enjoy/like the least.
- How have you worked to tailor the postures for different people?
- How has Zoom impacted engagement compared with face-to-face classes? Prompt for teacher engagement with the participants and vice versa.
- How has Zoom impacted communication compared with face-to-face classes? Prompt for teacher communication with the participants and vice versa.
- How does Gentle Years Yoga teaching differ from how you would usually address a mixed ability class? OR How does Gentle Years Yoga differ from other physically based classes you have attended?

### Data analysis

As per robust qualitative^35^ and longitudinal^38^ methodology, data analysis was iterative throughout the interview period. Data analysis was conducted by the process evaluation researchers (LWa and TR) according to the theories of thematic analysis.^39^ Analysis was inductive and responsive to the data, with no preconceived thematic framework.

Pen portraits were first written, integrating field notes with the interview transcripts to provide context to data. The data were then transferred to Excel spreadsheets and thematically grouped.^39^ Finally, themes were compared across subgroups of online and face-to-face participants and yoga teachers to determine if they were universal or context-dependent. Ongoing findings were presented at trial management and data committee meetings, enabling their input into refining the interview schedules and providing feedback on themes.

## Results

### Participant demographics

25 yoga participants (N=18 attended online yoga classes; N=14 completed longitudinal interviews) and eleven yoga teachers (N=3 delivered both online and face-to-face intervention; N=3 completed longitudinal interviews) took part in the interviews. Interviews covered the period from week 4 of the 12-week intervention to 36 weeks postintervention and ranged in duration from 15 to 98 min.

Participants were aged 66–91 years (mean age 74 years), predominantly white British (N=22, 88%) and female (56%), with IMD scores ranging from 1 to 10. Number of self-reported chronic health conditions ranged from 2 to 8 (mean 4.5 conditions per participant); most commonly osteoarthritis (N=15, 60%), cardiovascular disease (N=14, 56%), sensory conditions (N=9, 36%) and depression or anxiety (N=8, 32%). Yoga teachers were predominantly female (N=10, 91%), with yoga teaching experience ranging from 4 to 35 years across a range of yoga styles, and GYY teaching experience ranging from newly qualified to 5 years where stated.

### Teaching adaptations for optimal participant engagement

Teachers and participants drew on their experiences of both the GYY trial and their independent community-based activities to identify key features enhancing yoga accessibility for multimorbid and older adult cohorts. These were classified into six overarching categories covering 21 interconnected aspects of delivery, physical (yoga postures) and non-physical content, teaching style, communication and visibility. These aspects are outlined in detail below with supporting quotes and presented as an abridged teaching resource in table 1.

**Table 1:** 21-item teaching exemplar for optimising the safety and accessibility of face-to-face and online yoga classes for older adult and multimorbid populations

Category	Item	Teaching notes
Class delivery	Reduce the teacher-to-student ratio.	Lower teacher-to-student ratios offset the higher health and communication needs of older and multimorbid adults, by providing more time to adapt yoga practices to accommodate individual needs.
	Provide an individual session prior to the first group class.	Initial one-to-one session enables private discussion of individual’s health status and physical ability and opportunity to address any concerns regarding yoga.For online classes, additionally provide the opportunity to optimise chair placement for mutual visibility and conduct a visual risk assessment of the practice environment.
	Provide both face-to-face and online options.	Online classes are a preferred option for many individuals due to accommodating their health, social and work needs, and should be considered a delivery option in addition to face-to-face classes.
Physical content (yoga postures)	Keep physical content simple and repetitive.	Simple core practices of joint mobilisation and range of movement sequences are easily remembered and promote independent home practice.
	Choose physical content targeting functional independence.	Core physical content focused on joint mobilisation, range of movement and balance may maintain and improve physical function and activities of daily living.
	Choose physical content that retains mutual visibility.*	Focus on chair-based and standing poses, as individuals may not be easily visible on screen when doing mat-based poses.
Non-physical content	Incorporate breathing techniques.	Integrate simple breath awareness techniques, coordinated with physical postures, throughout the class, emphasising their benefit for biopsychosocial health management.
	Explain philosophical principles using universal rather than yoga concepts.	Enhance inclusivity of personal beliefs by highlighting the commonality between different belief systems and practices. For example, non-harm and treating others with kindness or mindfulness meditation and contemplative prayer.
	Teach chanting practices using neutral sounds rather than Sanskrit terms.	Enhance inclusivity of personal beliefs by neutralising the content of vocalisation practices. For example, replace Sanskrit terms with English words or vowel sounds.
Teaching style	Guide rather than instruct.	Individuals can better and more safely adapt yoga practices to their ability if provided with a mindful, educational approach to how a posture should feel within the body, rather than positional instruction of how it should look.
	Slow down and allow time to process the practice.	Provide individuals with time to experience the form and impact of the practices, to support them working safely within their physical limits and to develop awareness of their emotional responses.
	Bring energy to the class.	Bring more energy to your teaching style to counter potential low mood and limited feedback from individuals with higher or multiple health needs.
	Increase visual demonstration of postures.	Provide more visual cues than you would in a general yoga class, so individuals with difficulty processing speech always have a visual reference to follow.
Communication	Use simple and repetitive phrasing.	Use clear, simple, repetitive instruction to counter potential health issues such as impaired hearing or cognitive processing, and to aid retention for promoting independent home practice.
	Use individual names.	Using personalised rather than generic class feedback promotes a sense of inclusivity and tailored guidance. For online classes, it additionally provides individual reassurance of being seen in class.
	Use non-verbal communication.	Encourage the use of facial expressions and hand gestures as a proxy for verbal communication. This is equally relevant for both online delivery where muted microphones prevent real-time conversation, and face-to-face delivery where individuals may be uncomfortable with speaking in front of others.
	Interact with the camera.*	Move to and from the camera as a proxy for moving around a physical classroom. Provide close-up views of hand movements and breathing techniques and engage individuals in direct conversation using names and non-verbal feedback.
Visibility	Stand out from your background.	Have a plain background and floor surface, and wear contrasting coloured clothing. Use additional front and side lighting to supplement overhead lighting.
	Optimise positional information.	Wear light coloured, contrasting clothing on the upper and lower body to enhance positional information around the torso.Wear closer fitting clothing to enhance positional information of the upper and lower limbs.
	Reduce two-dimensional limitations.	Demonstrate a posture from multiple angles, to increase positional information of limb and torso placement.
	Optimise chair position prior to class.*	Use the pre-class individual online session to optimise mutual visibility and safety between teacher and class attendee via chair positioning.

* Denotes online classes only.

#### Class delivery

Teachers noted three pragmatic adaptations to class delivery for older adults and GYY-style classes. First, class size was reduced. Within the context of the GYY trial, a maximum of 15 participants were allocated to face-to-face classes and a maximum of 12 participants to online classes. Teachers noted their non-trial community-based GYY classes reflected these lower numbers compared with their general mat-based yoga classes which may have over 40 attendees. This reduced teacher-to-student ratio enabled teachers to provide more individual attention for adapting the yoga practices to an attendee’s specific health needs and physical capacity. Additionally, the lower online ratio enabled all participants to be visible on one screen:

I think, on the whole, I feel as though I can see them. I’m not so sure, if it was any more than 12 participants, because, I don’t know whether the squares would get smaller or the squares go up on a next screen, I don’t know. But, you know, all of mine are all on the same screen. Yoga teacher 1

Second, each online participant was provided with a one-to-one Zoom session with their teacher prior to the first group class. This session targeted older adults’ digital literacy and health needs, with the aim of optimising class accessibility and safety. Content included basic instruction in Zoom access and audiovisual controls and optimising mutual visibility of teacher and participant through the participant’s chair placement. Teachers also carried out a visual risk assessment of the participant’s practice environment and provided them an opportunity to privately discuss any health issues or concerns about taking part in the yoga classes. While only offered to online participants within the context of the GYY trial, both participants and teachers noted these individual sessions were a valuable part of both face-to-face and online community-based activities they either delivered or attended:

It was a great way to get people to do their space, and to do positioning, and to risk assess their space. […] We also tested out how far away they could be to still see me and hear me. Yoga teacher 2

Finally, participants wanted a choice of delivery formats. Many teachers had transitioned their community yoga classes to online delivery as a temporary response to COVID-19 social restrictions, expecting to fully return to face-to-face delivery once restrictions were lifted. However, due to an ongoing demand for online delivery, many now provide both delivery options. This feedback mirrored the GYY trial participants, with many stating a preference for online over face-to-face delivery due to its convenience, accessibility and adaptability to their health, social and work needs:

I’ve given them the choice to go back to face-to-face, but they find the accessibility of online brilliant. Yoga teacher 3

#### Physical content (yoga postures)

Three adaptations to optimise the physical content of yoga for an older and/or multimorbid demographic were promoted. First, content was kept simple and repetitive. Teachers tended to focus on the progressive practice of core basic postures and breathing techniques across the 12-week programme rather than introduce new yoga practices at each class. This supported long-term sustainability; focussing on a key set of basic practices optimised participants’ recall for their independent home practice.

Second, and related to the above, was choice of content. Teachers purposefully chose content to maintain and improve physical function and independent activities of daily living, such as joint mobilisation, range of movement and balance. The pragmatic relevance of this content was corroborated by participants, who favoured these simple, repetitive techniques for both ease of practice, functional maintenance and acute symptom management:

As I said, things which sound quite silly, like co-ordination and balance—it’s like the simplest thing in the world isn’t it, standing up or whatever, but actually if they don’t work you just fall down and then you’re calling for the NHS to come and pick you up off the floor. All of those things, they sound so simple, but actually, they are literally the foundations, they’re fundamental. Yoga participant 1

Third, and specific to online classes, content was structured around ease of visibility. While mat-based postures are permissible in GYY classes, teachers deemed them unsuitable for an online delivery format due to the time involvement and potential safety risk of repositioning cameras and computer equipment, chairs and mats to ensure mutual visibility was maintained. As such, the physical content of the online GYY classes was based on seated or standing yoga poses only.

#### Non-physical content

##### Importance of breathing techniques

Feedback indicated the most rewarding, transformative and sustained aspect of the GYY programme was the breathing practices. While most participants were used to physically based activity, the simple act of observing the breath was novel and delineated yoga from exercise. Incorporating the above aspects of simple and repetitive content, participants noted the sense of awareness, focus and calm induced by simply being aware of their breath throughout the class:

It’s the simple things again. You’re aware—breathing in the air and it’s cold, or cooler, and it comes out warm. I mean, I’ve got to [early 70’s] years of age, I’ve never realised that before. Yoga participant 2

##### Philosophy and chanting

An area of disparity within and between yoga teachers and participants concerned the practices of yoga philosophy and chanting, and their potential conflict with personal beliefs. Opinions on the appropriateness of teaching yoga philosophy to an older adult cohort depended on the framework of delivery. While both teachers and participants agreed philosophy was integral to delineating yoga from exercise, it was also noted that many participants came to yoga purely for the physical practice, and/or with their own faith which they viewed as incongruent with certain yogic teachings. Accordingly, many teachers chose to leave philosophy content out of their classes or taught it in terms of universal rather than yoga concepts; for example, highlighting the commonality between different belief systems such as mindfulness meditation and contemplative prayer:

Because I know it’s a religious base, I think it’s Hinduism or something like that, I think, or one of those religions. I must admit, I can’t accept that side of things, for obvious reasons*.* Yoga participant 3

Yogic chanting garnered similarly diverse opinions, with some teachers viewing chanting as core content and others viewing its practice as potentially conflicting with attendees’ personal belief systems. This latter view was reflected in participant interviews and further confirmed in class observations where many participants chose to remain silent during chanting practices. An acceptable adaptation to neutralising the chanting practices simply involved replacing Sanskrit terms with non-denominational sounds:

I look at who’s in front of me, and if you’ve got 70, 80, and 90 year old Christian, generally women, even if they don’t go to church, I don’t think it’s appropriate for me to say, “Well, now we’re going to chant the Gayatri Mantra” [laughter]. I mean it’s just not appropriate. However, what I can say to them is, look, there are some things in yoga that are good, which are chanting, and one of them is, we can do a sort of a chant on sound. So, I use the vowels, so I use AEIOU. Yoga teacher 4

### Teaching style

Most of the trial teachers adapted their usual yoga teaching style when delivering classes to older adults. Four main techniques were used to provide suitable information to learn these novel practices and enable time for participants to process and reflect on the impact of their practice. All but one of these adaptations were independent of delivery format.

First, teachers adapted their wording to guide rather than instruct participants, thus delivering a more mindful, educational approach to yoga practice as opposed to a didactic directive one. Participants noted this provided the opportunity to embody a version of a posture that best suited their unique physical ability, rather than copying a preset ideal of what a posture should look like. Focus was thus directed to what a participant could achieve, rather than what they couldn’t:

And I think the thing that they appreciated more, one of the things they appreciated was, I was guiding them rather than telling them it had to be. A lot of them said they felt there was no pressure, that anything that they did was fine. Yoga teacher 5

Second, some teachers slowed down the pace of delivery and extended the rest periods between postures. They felt the resultant reduction in the number of postures deliverable in a single class was offset by the experiential and safety benefits gained from having time to explore the form and impact of the movements. Participants confirmed this and further noted the calming impact of slowing down and reflecting was a defining point between yoga and exercise:

Exercise classes are, oh, daunting, and wham bam sort of things, you know? They don’t have any calming aspect to them. But the yoga movements are slower and there’s a lot more focus on being calm and relaxed and things, you know, on closing your eyes and just feeling the movement and things. Yoga participant 4

Third, teachers needed to be empathetic and responsive to the physical, mental and social issues of older adults. Many stated they brought a higher level of energy to teaching GYY classes compared with general classes, to counteract potential low mood and lack of feedback they often experienced within this demographic. This was corroborated by participants, who directly related their experiences of the classes to the emotional connection they did, or did not, feel with the teacher:

I think, to be honest, it was the way [teacher] came across as well, so incredibly positive and caring, that, you know, I warmed to that very quickly. And that really did help my mood as well. Yoga participant 3

Lastly, teachers agreed on a need for increased demonstration of the physical postures, to maximise visual information for participants. This supplemented the often multistep verbal explanations when learning a new posture and was particularly relevant to online delivery where the two-dimensional nature of a computer screen restricted positional information:

I think the difference in teaching is that in our general classes we perhaps wouldn’t, or don’t demonstrate the whole time. In Gentle Years Yoga it’s important to demonstrate the whole time because people need the visual cue to follow. Yoga teacher 6

### Communication adaptations

Cognitive, visual and hearing impairments within older adult cohorts necessitated four communication adaptations for GYY delivery. First, understanding of the yoga practices was enhanced through language choice. Participants preferred the short, repetitive and consistent phrasing used by some teachers compared with the more verbose descriptions used by others. Simple, stepwise instruction helped participants learn a new posture or breathing technique and sustain that knowledge for self-directed practice following the trial:

There is a little bit of a knack, I think, to teaching on Zoom. And I think one of them is to go a little bit slower. Do you know what I mean? And I think to keep the language consistent and simple. So trying to be consistent with the language that you’re using. Yoga teacher 1

Second, and divisive among yoga teachers, was the use of participant names. While some teachers felt using an individual’s name supported inclusivity and progression, others strongly felt that it singled individuals out and only gave generic group feedback. Participants, however, had a clear preference—use their names. Particularly for online classes, hearing their name counteracted the lack of real-time communication by confirming the teacher could see them and was guiding them to practice both safety and correctly:

I know that, in class, some of my own students are very worried about picking on people, and I teach them that it’s not picking. They want to be helped, so it’s a positive thing. Yoga teacher 7

Third, non-verbal communication was effectively used to both give and receive participant feedback. Class observations confirmed interview feedback that participants felt more engaged in classes where the yoga teachers encouraged them to use facial expressions and hand gestures as a proxy for verbal communication. This form of feedback was equally relevant for both online delivery where muted microphones prevent real-time conversation, and face-to-face delivery for participants uncomfortable with speaking publicly in a group.

A final communication adaptation, specific to online delivery, involved interactive engagement with the camera. Participants preferred when teachers moved back and forth to the camera, describing it as a proxy for them walking around the group in a physical classroom. Interactions included providing close-up demonstrations of physical movements and breathing practices, and directly engaging with participants using previously noted verbal and non-verbal communication techniques:

Well yeah, because, you know, [teacher] will come and sit at the front for part of it and say, “I’m doing this.” And then say, “I’m moving to the back here, I’m going to do this. Can you hear me?” Then [teacher] might say, “[Name], are you okay?” Or, “[Name], are you okay? [Name], are you okay?” or something like that. So, we’re all on mute, but it’s—And then [teacher] say, you know, “Thumbs up.” So, you do feel you’re part of a group, but not sort of seeing the group. Yoga participant 2

### Visibility

Specific to GYY online delivery was optimising visibility—both the teachers’ view of their participants and vice versa. As mentioned previously, visibility was initially addressed via chair positioning in the preclass Zoom session. Additionally, interviews and observations noted four simple adaptations to maximise yoga teacher visibility through environmental and clothing choice. First, teachers needed to stand out from their background, with some teachers hanging a plain sheet or screen on the wall behind them. Second, the colour of their clothing needed to contrast with the background. Third, their foot position needed to be clearly visible on the floor, achieved by using a contrasting coloured yoga mat. Fourthly, teachers needed to be well lit from the front and side, with many purchasing free-standing studio-style lamps to supplement in situ overhead lighting:

I got myself a portable blue screen which was really cheap. And I also found you got to look at the contrast as well, so that’s why I have two mats on the floor. It’s just things you just never thought you’d need. Yoga teacher 8

Clothing choice further impacted teachers’ positional information. Visibility was enhanced when teachers wore contrasting, light coloured clothing on the upper and lower body; conversely, observations noted monotone dark clothing reduced positional information of the trunk (from shoulder to upper thigh region) in both seated and standing positions. Furthermore, clothing that was closer fitting provided better positional information of a teacher’s arms and legs than looser clothing.

Positional visibility was further enhanced by adaptations to counter the two-dimensional nature of a computer screen. This was achieved by the yoga teacher demonstrating a posture from multiple angles so participants could better determine limb and torso placement:

I also tell them I’m going to move around a lot, “I don't expect you to keep moving around, but I’m going to move my chair forward/back, I’m going to demonstrate some stuff on the side so you can see what’s happening”. Yoga teacher 8

## Discussion

Yoga, by nature, is an adaptable practice.^10^ However, both older adult and multimorbid cohorts bring to a yoga class aspects of health and ageing not necessarily covered in basic yoga training.^27^ This paper presents a teaching exemplar for optimising the safe and inclusive delivery of chair-based yoga to older adults with multiple long-term health conditions, based on the experiences of yoga teachers and participants from the GYY trial. Synthesised qualitative feedback has informed a 21-item list covering broad aspects of face-to-face and online teaching, including class delivery, yoga content, teaching style, communication adaptations and visibility.

Paramount to the delivery of yoga for older adults is safety. While evidence presents yoga as a safe form of exercise,^22^ yoga-related injuries are highest in the older adult age group.^40^ As many older adults come to yoga from a sedentary lifestyle,^41^ even relatively simple movements may be challenging or unsafe.^27^ A recent ethnographic study noted that reducing older adults’ risks during yoga requires a high level of pedagogical skills due to the diverse physical abilities of this age cohort.^42^ Further modifications associated with the two-dimensional nature inherent to the online delivery of yoga were noted from the Successful AGEing (SAGE) trial, investigating yoga for falls reduction in older adults.^24 43^

The GYY yoga teachers and participants raised multiple items addressing safety, confirming these previous research studies, and highlighting the role of communication and visibility in promoting a safe yoga environment. Notably, safety considerations should precede the first yoga class. The GYY yoga teachers and participants supported the use of individual preclass sessions to enable teachers to gain the health information needed to individually tailor class content.^42 43^ For online yoga delivery, these sessions additionally provide the opportunity to address technology issues and counter visibility and communication restrictions inherent to online delivery by optimising the setup of the home environment and assessing the safety and appropriateness of props.^24 43^ Once classes begin, relevant communication adaptations include clear simple instruction,^43 44^ a slower pace of delivery,^43^ and the use of individual names.^43^ Visibility adjustments include enhancing positional information of limb and torso placement, the area where yoga-related injuries most commonly occur,^40^ through demonstration of postures from multiple angles,^43 44^ and pragmatic choices around clothing and background to maximise the contrast of the teacher from their background.

Inclusivity of yoga for older adults is, by default, enhanced by these safety considerations. It is further supported in this teaching exemplar through the choice of content. Breathing techniques are key to the experience of yoga for older adults,^33^ yet they may comprise as little as 15% of class time.^11^ Our teaching exemplar supports previous recommendations for the inclusion of breathing practices in yoga for older adults.^42 43^ Inclusion of individual beliefs^42^ is also supported, through items suggesting philosophical and chanting content be delivered in neutral terms and universal concepts acceptable to all.

In addition to supporting previous research, our teaching exemplar extends the evidence base by providing new items for the use of yoga as a health-management tool for older adults. Teacher and participant feedback promotes the delivery of yoga with a more energetic teaching style, to counter potential low mood and limited feedback from individuals with higher or multiple health needs. For online delivery, this includes purposeful use of non-verbal communication such as hand gestures and facial expressions to engage participants in two-way communication, and dynamic interaction with the camera through moving back and forward to show close-up and full body demonstrations. Additionally, this teaching exemplar promotes physical content based on simple core practices of joint mobilisation, balance and range of movement sequences that target functional independence. Participant feedback advises that simple, repetitive and relevant content is easily remembered and promotes independent home practice.

### Future implementation

The high prevalence of older adults not meeting activity guidelines,^45^ combined with the negative health implications of an increasingly sedentary lifestyle,^46 47^ highlights the need for new ways to engage older adults in physically based activities. Outcomes from the GYY trial indicate older adults view both face-to-face and online delivery of chair-based yoga as safe, acceptable, and adaptable to their acute health needs and lifestyles.^33 34^ Together with the expanding evidence base for the effectiveness of yoga for a range of chronic health conditions common to older adults, this situates yoga as a key option for activity engagement.

However, while the practice of yoga in the general population increases,^9^ key issues remain regarding the delivery of yoga to an increasing multimorbid older adult population.^2 48^ Yoga teachers and training courses are often unregulated, and a lack of specialised knowledge of the physiological implications of ageing, multimorbidity and polypharmacy may result in inappropriate content being included in yoga classes delivered to this higher-risk demographic.^27^ This, together with a lack of evidence-based guidance for the application of yoga to specific chronic health conditions, has led to a call for specialised training courses to improve teachers’ knowledge of both clinical conditions and the safe modification of yoga practices in their management.^49^ This process has begun, with research into tailoring yoga content to older populations,^26 28^ and targeted programmes such as the Ofqual-regulated GYY programme^32^ and the Relax Into Yoga for Seniors programme.^27^ This publication’s 21-item guidance further provides insight into the delivery of these yoga practices in a way that both promotes safety and sustains self-practice of yoga as a health management tool beyond the classroom and into everyday life.

A future direction for the implementation of this research involves the link between health practitioners’ knowledge of the suitability of yoga for older adults and their support of its uptake for their client’s health management. While the use of yoga increases among the general population, acceptance and use of complementary and complementary medicine (CAM) approaches such as yoga varies across medical specialties.^50^ Many health practitioners are unfamiliar with the evidence base for CAM effectiveness, and knowledge of accredited yoga programmes is low even among healthcare practitioners who themselves practice yoga.^51^ These factors, together with a lack of knowledge around the provision of appropriate CAM services within the community,^51 52^ are noted as significant barriers to recommending yoga as a health management tool to patients.

Moving forward, addressing calls for individuals’ active participation in their health management^5^ requires deliberate and collaborative conversations between researchers, healthcare practitioners, yoga organisations and the public to develop evidence-based yoga programmes and regulated teacher training programmes promoting yoga as a safe and effective lifestyle behaviour.

### Strengths and limitations

This study has provided an in-depth exploration of effective teaching techniques through engaging the key stakeholders of a yoga class, the teachers and the participants. While generalisations are limited in qualitative research, judicious use of qualitative methodology ensured a broad spectrum of the older adult demographic was given a voice, enabling insight into how factors including age, health status and lifestyle impact the role of yoga for health management. By comparing feedback among and between these stakeholder groups, areas of disparity have been highlighted in relation to class content and teaching style, and clear preferences stated for an effective and inclusive yoga experience. Additionally, while this guidance is based on feedback from an older adult demographic, given the prevalence of chronic health conditions in the general population, it may prove beneficial to any yoga teacher delivering public classes.

Findings from this study should be considered in the context of the participant cohort, and with recognition that the 21-item teaching exemplar was developed from chair-based rather than mat-based yoga delivery. The primary cohort limitation is related to ethnicity. While participants for this qualitative study were purposely selected to represent the ethnic diversity of the main trial cohort, it is noted that opportunities for this were limited due to the trial’s majority White British population.^30^ Another limitation relates to positive selection bias and sample size. While purposive sampling and meaning saturation techniques minimised sampling bias within the trial context, it is acknowledged that the size of embedded qualitative cohorts is inherently small in comparison to the main trial cohorts they are sampled from. Also, the views of trial participants may not reflect the wider community-based cohort of older adults with multimorbidity, or of those who declined to participate in the GYY trial. These limitations could be addressed in future research through exploring the use of the 21-item teaching exemplar among a more ethnically diverse cohort and conducting focus groups to investigate if these adaptations to optimise participant safety and inclusion may encourage more older adults to try yoga for their health and well-being. Additionally, the application of this teaching exemplar to mat-based classes, and yoga teacher feedback on its usefulness, is a pragmatic next step for exploration.

## Conclusions

The practice of yoga is both increasingly popular among older adults and evidenced as effective for the management of chronic health conditions common to this demographic. This study advances the evidence base for optimising the delivery of yoga to an older adult cohort, supplementing existing recommendations on the adaptation of yoga postures to reduce risk and enhance physical outcomes. Our 21-item list, based on feedback from yoga teachers and participants involved in the GYY trial, provides pragmatic guidance to yoga teachers for optimising the safe and accessible delivery of both face-to-face and online chair-based yoga. This pragmatic guidance covers aspects of class setup, recommendations for core, simple and repetitive content which is inclusive of personal beliefs and physical ability, and key communication techniques to offset age-related and online audiovisual restrictions. Future research directions for optimising the role of yoga as a health management tool for the growing older adult population include health practitioner education on yoga’s safety and effectiveness, and the development of specialised yoga training courses targeting older adults’ needs and common health issues.

## Data Availability

No data are available.
