# Supplementary material for: Yoga for older adults with multimorbidity: teaching insights for optimising participant safety and inclusion from the process evaluation of the Gentle Years Yoga trial
Source: BMJ Open. 2025 Jun 27;15(6):e097472. doi: 10.1136/bmjopen-2024-097472 (PMC12207179; doi:10.1136/bmjopen-2024-097472)
Supplement: Supplementary file 1 [file bmjopen-2024-097472.reviewer_comments.pdf]

PEER REVIEW HISTORY

BMJ Open publishes all reviews undertaken for accepted manuscripts. Reviewers are asked to complete a checklist review form and are provided with free text boxes to elaborate on their assessment. These free text comments are reproduced below.

ARTICLE DETAILS

Title (Provisional)

Yoga for older adults with multimorbidity: teaching insights for optimising participant safety and inclusion from the process evaluation of the Gentle Years Yoga trial

Authors

Ward, Lesley; Wiley, Laura; Rose, Fiona; Howsam, Jenny; Bissell, Laura; Tew, Garry; Rapley, Tim

---

VERSION 1 - REVIEW

**Reviewer**	**1**
**Name**	**Majumdar, Vijaya**
**Affiliation**	**Swami Vivekananda Yoga Anusandhana Samsthana, Molecular Bioscience**
**Date**	**22-Feb-2025**
**COI**	**None**

The manuscript addressed key issues pertaining to optimisation of participant safety and inclusion of multimorbid older population. The inclusion of teacher's insights is the key highlight of the study.

I have only one minor suggestion, if authors could consider the inclusion of participants of majorly white British decent as a limitation.

**Reviewer**	**2**
**Name**	**Pathania, Monika**
**Affiliation**	**AIIMS Rishikesh, Internal Medicine**
**Date**	**15-Mar-2025**
**COI**	**None**

The article is well timed as per the trend towards using yoga, however not much has been written about yoga in the introduction. yoga includes eight limbs and breathing techniques or pranayama is one of them. The author must give authentic introduction as per the

authentic text of yoga , naming the asanas in thier original name along with its name in english will only do justice if you want to research on yoga.

**Reviewer**	**3**
**Name**	**Wister, Andrew**
**Affiliation**	**Simon Fraser University, Gerontology**
**Date**	**20-Mar-2025**
**COI**	**None**

This is an important addition to the literature pertaining to yoga training for older adults and those with multimorbidity. The manuscript is clearly written and well-organized. However, there are some minor issues that require attention prior to publication in my opinion.

1) The Introduction is very brief and does not situate the study into the existing literature, Some prior work provides a rationale for this study and the focus on older adults, which should be incorporated into an expanded Introduction. I have mentioned a couple of studies below, and there are others that are not fully utilized from the authors' reference list.

Chen, K. M., Tseng, W. S, Ting, L. F., & Huang, G. F. (2007). Development and evaluation of a yoga exercise programme for older adults. Journal of Advanced Nursing,

57 (4), 432 – 441 .

Chen, K., Chen, M., Hong, S. M., Chao, H., Lin, H., & Li, C.(2008). Physical fitness of older adults in senior activity centres after 24-week silver yoga exercises. The Journal of

Clinical Nursing , 17 , 2634 – 2646 .

Cho, H. K., Moon, W., & Kim, J. (2015). Effects of yoga on stress and inflammatory factors in patients with chronic low back pain: A non-randomized controlled study.

European Journal of Integrative Medicine, 7 (2), 118 – 123 .

Patel, N., Newstead, A., & Ferrer, R. (2012). The Effects of Yoga on Physical Functioning

and Health Related Quality of Life in Older Adults: A Systematic Review and Meta-Analysis. THE JOURNAL OF ALTERNATIVE AND COMPLEMENTARY MEDICINE

Volume 18, Number 10, 2012, pp. 1–15.DOI: 10.1089/acm.2011.0473

Wertman, A., Wister, A., & Mitchell, B. (2016). On and off the mat: Yoga experiences of middle-aged and older adults. Canadian Journal on Aging, 35(2): 190-205.doi:10.1017/S0714980816000155.

2) Methods & results are well done.

3) Discussion - I believe that the discussion section does not do the study justice. I would like to see some strengthening of the results and situating these into the current knowledge and

literature. For example, the section that discusses three key messages misses many important areas to guide yoga teachers, for instance, the importance of adapting programs to meet the unique health conditions, preferences, and needs of older adult populations. There are several others that fall out of the results, but are not mentioned in the discussion. Furthermore, what gaps are filled and what needs to be done?

4) The Strengths and Limitations section is weak. For example, there should be articulation of selection effects and social desirability that may have skewed the results to some degree. Other limitations related to the small n size should be exposed to the reader.

5) The Conclusion could be stated in more forthright wording exemplifying the contributions of this important work to the audience.

VERSION 1 - AUTHOR RESPONSE

**Source**	**Reviewer comment**	**Author response**
Reviewer 1	I have only one minor suggestion, if authors could consider the inclusion of participants of majorly white British decent as a limitation.	I have added three sentences and a reference to address this limitation. I have also rearranged this section into two paragraphs, so that this limitation is now highlighted in a separate paragraph of its own.
Reviewer 2	The article is well timed as per the trend towards using yoga, however not much has been written about yoga in the introduction. yoga includes eight limbs and breathing techniques or pranayama is one of them. The author must give authentic introduction as per the authentic text of yoga , naming the asanas in their original name along with its name in english will only do justice if you want to research on yoga.	Thank you for this direction as to the depth of background information to include. I have added a paragraph to the Introduction to give context to the authentic place of yoga within Indian philosophy, have used both English and Sanskrit terms to describe the eight limbs, and have included three new references. I have then made a separate paragraph to discuss the separate practice of yoga in the West, differences in focus, changing demographics of users, and added additional references.
Reviewer 3	The Introduction is very brief and does not situate the study into the existing literature. Some prior work provides a rationale for this study and the focus on older adults, which should be incorporated into an expanded Introduction. I have mentioned a couple of studies below, and there are others that are not fully utilized from the authors' reference list.<br>* Chen, K. M., Tseng, W. S, Ting, L. F., & Huang, G. F. (2007). Development and evaluation of a yoga exercise programme for older adults. Journal of Advanced Nursing, 57 (4), 432 – 441 .<br>* Chen, K., Chen, M., Hong, S. M., Chao, H., Lin, H., & Li, C.(2008). Physical fitness of older adults in senior activity centres after 24-week silver yoga exercises.	Thank you for your feedback. I have undertaken a major revision of the Introduction, based on comments from yourself and another reviewer, and doubled the length of this section.<br><br>I also note that the focus of this paper is around the development of delivery guidelines of yoga sessions for older adults, rather than yoga's effectiveness for older adults. To this end, I have now been able to reference two relevant publications from our Gentle Years Yoga trial, that were under submission at the time this paper was

	The Journal of Clinical Nursing , 17 , 2634 – 2646 .<br>* Cho, H. K., Moon, W., & Kim, J. (2015). Effects of yoga on stress and inflammatory factors in patients with chronic low back pain: A non-randomized controlled study. European Journal of Integrative Medicine, 7 (2), 118 – 123 .<br>* Patel, N., Newstead, A., & Ferrer, R. (2012). The Effects of Yoga on Physical Functioning and Health Related Quality of Life in Older Adults: A Systematic Review and Meta-Analysis. THE JOURNAL OF ALTERNATIVE AND COMPLEMENTARY MEDICINE Volume 18, Number 10, 2012, pp. 1–15.DOI: 10.1089/acm.2011.0473<br>* Wertman, A., Wister, A., & Mitchell, B. (2016). On and off the mat: Yoga experiences of middle-aged and older adults. Canadian Journal on Aging, 35(2): 190-205.doi:10.1017/S0714980816000155.	being written, to signpost that the issues of both effectiveness of the GYY trial, and experiences of online delivery of yoga for older adults, have been reported elsewhere.<br><br>Regarding the references mentioned:<br>• when writing the original submission I had accessed the Chen et al references but had decided not to include them, as the research was 20 years old and either explorative (2007) or quasi-experimental (2008). However, I have now included the 2007 reference in regard the age-related adaptations they made to yoga content.<br>• I have chosen not to include the Cho (2015) reference, as this is the wrong age demographic and just a single study; I have instead cited a systematic review for chronic low back pain (Anheyer et al, 2022).<br>• I have chosen not to include the Patel reference, as I have cited more recent individual systematic reviews of specific health conditions, which show higher methodological quality than those included in the Patel review.<br>• I have included the Wertman et al reference in multiple places.
Reviewer 3	Discussion - I believe that the discussion section does not do the study justice. I would like to see some strengthening of the results and situating these into the current knowledge and literature. For example, the section that discusses three key messages misses many important areas to guide yoga teachers, for instance, the importance of adapting programs to meet the unique health conditions, preferences, and needs of older adult populations. There are several others that fall out of the results, but are not mentioned in the discussion. Furthermore, what gaps are filled and what needs to be done?	Thank you for this advice. I have completely rewritten the Discussion, which is now almost twice as long. Our results are now better situated in the literature, with multiple new references included, and I have clarified where our findings support previous research and where they advance it by adding new insights. I have rewritten the section on future directions, to show more broadly where the barriers to implementation of yoga for older adults are (lack of specialised training, and lack of health practitioner awareness of the evidence base) and suggested directions for future research.
Reviewer 3	Strengths and Limitations section is weak. For example, there should be articulation of selection effects and social desirability that may have skewed	I thank you for this feedback, and acknowledge that improvements in the limitations section have also been

	the results to some degree. Other limitations related to the small n size should be exposed to the reader.	requested by another reviewer and the editor. To address this, I have added a new paragraph addressing limitations related to both the participant cohort (lack of ethnic diversity, sample size, selection bias) and format of the GYY programme (chair-based, not mat-based). I have then suggested ways to address these limitations in the next stages of research, including feedback on the pragmatic use of these teaching recommendations within the yoga teaching community.
Reviewer 3	Conclusion could be stated in more forthright wording exemplifying the contributions of this important work to the audience.	I have rewritten and added to the conclusion. It is now written in an active voice, provides a concise overview of the 21-item list, and provides direction for future research.
